# Barrett’s esophagus and esophageal cancer: Links to microbes and the microbiome

**DOI:** 10.1371/journal.ppat.1007384

**Published:** 2018-12-20

**Authors:** Teminioluwa A. Ajayi, Sarah Cantrell, Ashley Spann, Katherine S. Garman

**Affiliations:** 1 School of Medicine, Duke University, Durham, North Carolina, United States of America; 2 Department of Medicine, Duke University, Durham, North Carolina, United States of America; 3 Division of Gastroenterology, Department of Medicine, Duke University, Durham, North Carolina, United States of America; 4 Durham Veterans Affairs Health Care System, Durham, North Carolina, United States of America; Tufts Univ School of Medicine, UNITED STATES

## Introduction

Esophageal cancer represents a major problem globally, with an estimated 398,000 esophageal squamous cell cancers (ESCC) and 52,000 esophageal adenocarcinomas (EAC) diagnosed worldwide in 2012 [1]. In the United States, EAC is more common than ESCC, and EAC in the US increased dramatically between 1997 and 2006 before stabilizing [2]. Furthermore, esophageal cancer remains particularly deadly, with a five-year survival rate of less than 20% [3]. EAC is thought to arise from Barrett’s esophagus (BE), a metaplastic differentiation from normal squamous epithelium to a columnar phenotype. Accepted risk factors for BE and progression to EAC include age, male sex, obesity, gastroesophageal reflux disease (GERD), and smoking [4, 5]. Unfortunately, less is known about the association between esophageal dysbiosis, inflammation, and the pathogenesis of BE and EAC. Most of the existing data on esophageal microbiota in BE and esophageal cancer are derived from small cross-sectional studies with limited information on causality. As such, this represents an important area for future research because changes in the esophageal microbiota may relate to modifiable factors such as antibiotic use and proton pump inhibitors (PPIs).

The esophageal microbiota was initially thought to be primarily related to transient esophageal exposure to the oral microbiota [6], but more recently, culture-independent studies have shown the esophageal microbiota to represent its own niche [7]. In addition to swallowed oral bacteria, the esophagus is also exposed to refluxed gastric microbes, and while the esophageal microbiota resembles both oral and gastric microbiota, it is equal to neither [7, 8]. This review provides an overview of differences that have been described between the normal esophageal microbiota, BE, and EAC. We also discuss novel approaches to esophageal microbial sampling and consider clinical exposures that may alter the esophageal microbiota.

## Methods

A comprehensive literature review was conducted by a professional librarian (SC) in February 2018. Three biomedical databases—MEDLINE (PubMed), Embase (Elsevier), and Web of Science—were searched for relevant studies. The primary search strategy was created in PubMed and included a combination of text word and Medical Subject Heading (MeSH) terms. Primary search concepts included microbiota or microbiome, as well as BE or esophageal neoplasms. The search was then translated to the two additional databases, Embase and Web of Science. The full search strategy can be found in the Supporting Information S1–S3 Tables. Citations were managed using EndNote X8. The queries retrieved 658 citations with 239 duplicate citations identified using EndNote, leaving 419 citations for review. The itemized search strategy can be seen in Supporting Information S1–S3 Tables. 419 citations were reviewed manually by two authors (TAA and KSG) for relevance to esophageal microbiome, BE, and/or esophageal cancer. This process resulted in selection of 16 primary papers that included primary data relevant to the topic, and these are listed in Supporting Information S4 Table.

### Normal esophagus is predominantly colonized by *Streptococcus* while esophagitis and BE are associated with gram-negative bacteria

The microbial flora of the esophagus was described in 1998 using cultures of aspirated esophageal washings, with 66.7% of samples yielding a positive culture with *Streptococcus viridans* and group D *Streptococcus* [6]. However, this study was not performed in healthy patients because all subjects had presented for endoscopy to evaluate symptoms of dyspepsia but were found to be without obvious infection or gross esophageal abnormality [6]. Pei and colleagues provided the first non-culture–dependent description of esophageal bacteria using broad-range 16S ribosomal DNA (rDNA) gene clone sequencing [9]. Six distinct phyla—including *Firmicutes*, *Bacteroides*, *Actinobacteria*, *Proteobacteria*, and *Fusobacteria*—were identified in this study, and *Streptococcus* was found to be the most prevalent genus (38.6%) [9]. However, this study was performed in patients who presented for upper endoscopy because of gastrointestinal symptoms [9]. While the esophageal histology was normal, the study was limited to four Hispanic adults with gastrointestinal symptoms. Thus, while this small study provides our best estimate of “normal” esophageal microbiota, it does not provide a definitive description of a normal esophageal microbiota in healthy asymptomatic individuals.

In a histologic analysis of patients with GERD or BE, Gram stains were performed on esophageal endoscopic biopsies from 10 patients with normal histology, 10 with GERD characterized by erosive esophagitis, 10 with nondysplastic BE, and 17 with dysplastic BE (10 low-grade, 7 high-grade dysplasia) [10]. Of note, PPI use was common across all study groups [10]. Overall, the histologic bacterial score, defined as a score from 0–4 assigned by a pathologist unaware of clinical status and ranging from 0 = none to 4 = more than four clusters or numerous scattered bacteria, was higher in patients with BE compared to normal histology, with the highest scores seen with patients with high-grade dysplasia [10]. The localization of bacterial organisms on the mucosal surface of the esophageal epithelium was affirmed in studies of histologic staining by other groups [9, 11, 12].

In order to characterize the bacteria associated with different types of esophageal histology, Yang and colleagues performed 16S rDNA gene sequencing on esophageal mucosal biopsies and found that when compared with samples of normal esophagus, samples from areas of esophagitis and BE had reduced *Streptococcus* and increased gram-negative anaerobes and microaerophiles [13]. When patterns of esophageal microbiota were classified into two groups, the esophagitis and BE samples were associated with the “type II microbiome” with an odds ratio (OR) of 15.4 (95% CI 1.5–161.0) for esophagitis and OR 16.5 (95% CI 1.5–183.1) for BE. However, the sensitivity of this classifier was only 58%–60%. While the Yang paper is an often-cited, landmark study, it is important to note that at least two of the “normal” samples were from patients with BE and/or a tumor, and all of the “normal” samples were obtained from patients with an indication for upper endoscopy such as blood loss, heartburn, BE, or nausea [13]. In addition, as reported in Supporting Information S4 Table, esophagitis was defined broadly, spanning a range of indications for the upper endoscopy and relying upon the presence of at least 10 lymphocytes per high power field (found in all 12 patients in the esophagitis group); in five of the esophagitis cases, eosinophils were identified, and in one of the cases, polymorphonuclear cells were present as well [13]. The reduction in *Streptococcus* associated with BE was also found in a study performed by Liu and colleagues in which patients presenting for upper endoscopy were defined both endoscopically and histologically as normal esophagus, reflux esophagitis, or BE [14]. In addition, *Veillonella*, *Fusobacterium*, and *Neisseria* were found in some patients with reflux esophagitis and BE but not in normal samples [14]. Using culture techniques, *Campylobacter* was identified in about 50% of both reflux esophagitis and BE patients and was associated with increased interleukin-18 (IL-18) [12, 15]. It has been suggested that gram-negative bacteria may increase Toll-like receptor (TLR) signaling and expression of downstream inflammatory cytokines that cause disease, but more direct evidence is needed regarding the functional significance of these organisms in esophageal metaplasia. Indeed, because of the heterogeneity of these patient populations, the lack of true-normal heathy control patients, and the cross-sectional nature of these data, these remain associations, and causality cannot be implied.

### EAC has been associated with specific gram-negative organisms including *Escherichia coli* and *Fusobacterium nucleatum*

As noted above, GERD with esophagitis and BE have been associated with a more gram-negative esophageal microbiota, and it appears that this persists in the transition to esophageal cancer. Human esophageal samples from a small study of 28 patients revealed *Escherichia coli* in only BE and EAC but not in the normal esophagus [16]. A rat model of EAC was reported by Zaidi and colleagues in which esophagojejunostomy was performed, allowing direct exposure of jejunal contents to the esophagus [16]. The notable limitations to the rat model have been widely reported [17] and include 1) the induced esophageal injury via the anastomosis differs physiologically from the (gastric) reflux in humans, 2) the “BE” noted in the rat model may be due to migration of intestinal cells into the esophagus, and 3) mortality in the rats is high (about 30%). In the Zaidi study, surviving animals were followed for 40 weeks [16]. The final analysis included five EACs from the surgery group (*n* = 37) and four control animals [16]. *E*. *coli* was present in three of five “BE” samples from the rat, one normal rat control, and all five EAC samples [16]. Statistical analysis of the study was quite limited, making it difficult to interpret the bacterial findings. Gene expression analysis did include statistical analysis and suggested an up-regulation of TLR signaling was present [16]. Indeed, lipopolysaccharides present in gram-negative bacteria activate TLRs and promote secretions of proinflammatory cytokines and activation of nuclear factor κ-light-chain enhancer of activated B cells (NF-κB) signaling [18, 19]. In patients with BE and BE-associated EAC, studies have shown progressive expression of NF-κB through BE oncogenesis and a correlation between NF-κB expression in EAC and response to neoadjuvant chemotherapy and radiotherapy [20].

Another gram-negative species, *Fusobacterium nucleatum*, has been associated with gastrointestinal neoplasia, including esophageal cancer. *F*. *nucleatum* was associated with 23% of esophageal cancers (EAC and squamous cell), and the presence of *F*. *nucleatum* was a poor prognostic factor correlating with advanced tumor stage and poorer cancer-specific and overall survival [21]. Highly associated with periodontal disease and known to adhere to and invade epithelium, *F*. *nucleatum* may promote esophageal carcinogenesis through several mechanisms, allowing for tumor evasion of the immune system including activation of beta-catenin signaling, induction of cytokine secretion, and immune inhibition via T cell suppression [21]. While outside the scope of this review, it is worth noting that several studies have demonstrated a causal and mechanistic relationship between *F*. *nucleatum* and colorectal carcinogenesis. *F*. *nucleatum* increases tumor formation in mouse models of colon tumor development and is associated with local expansion of myeloid-derived immune cells [22]. In colorectal cancer cell culture models and xenograft experiments, *F*. *nucleatum* has been shown to bind epithelial cadherin (E cadherin) and activate beta-catenin, triggering local inflammation and growth [23]. TLR4 activation, NF-κB signaling, and microRNA-21 (miR-21) expression are induced by *F*. *nucleatum*, which is also associated with poor overall survival in human colorectal cancer patients [24]. Functional studies of the role of *F*. *nucleatum* in the esophagus will likely provide important insights into esophageal carcinogenesis.

Alterations in oral microbiota may also be associated with esophageal cancer risk. In two large cancer screening and prevention cohorts, a nested case-control study was performed on 81 EAC cases and 160 matched controls [25]. Oral wash samples were collected, and the oral anaerobic gram-negative bacteria *Tannerella forsythia* was associated with a small increased risk of EAC (OR 1.21, 95% CI 1.01–1.46, *p* = 0.04). *Actinomyces cardiffensis*, *Selenomonas* oral taxon 134, and *Veillonella* oral taxon 917 were also associated with increased risk of EAC, while other organisms from the oral microbiota such as *Prevotella nanceiensis* and *Streptococcus pneumoniae* were associated with protective effects [25].

In the stomach, while *Helicobacter pylori* is a well-established risk factor for gastric cancer, a recent meta-analysis revealed that *H*. *pylori* infection is protective against EAC (OR 0.55, 95% CI 0.47–0.66) [26]; population-based case-control studies have supported this finding [27]. Several groups have identified *H*. *pylori* in esophageal tissue, but the clinical significance of this remains unknown [28, 29]. Rather than a direct mucosal effect of *H*. *pylori* in the esophagus, the protective effect of *H*. *pylori* infection may relate to the reduced levels of gastric acid production and less acid-related esophageal injury.

In addition, alterations in the gastric microbiota have been associated with esophageal dysplasia and ESCC. Increased abundance of certain species in the gastric fundus, such as *Clostridiales* and *Erysipelotrichaceae* from the phylum Firmicutes, were associated with esophageal squamous dysplasia and ESCC in a case-control study performed in Iran [30].

Finally, to date, the emphasis on the esophageal microbiota has been on bacteria. However, in some series, *Candida albicans* and *C*. *glabrata* were detected in more than half of all EAC samples, but our understanding of the role for fungi in the pathogenesis of esophageal neoplasia remains extremely limited at this time [16]. In the future, newer approaches that more broadly measure esophageal viruses, fungi, and bacteria are likely to provide a more complete characterization of these organisms in both the normal human esophagus and in disease.

### Penicillin exposure has been associated with increased risk of esophageal cancer

Given the associations between esophageal dysbiosis and disease, factors that alter the esophageal microbiota are important to consider. Specifically, antibiotic use represents a common clinical exposure. However, the role of antibiotics in the pathogenesis of BE and esophageal cancer remains an ongoing topic for investigation, and no definitive studies have been performed. Using a population-based cohort in the UK, a recent epidemiologic case-control study demonstrated a dose-dependent association between penicillin exposure and increased risk of various cancers, including esophageal cancer [31]. However, the type of esophageal cancer (EAC versus ESCC) was not specified, and causality should not be inferred. It is possible that this increased cancer risk associated with penicillin use may result from a decrease in protective bacteria such as *Streptococcus* following antibiotic administration. In order to understand the relationship between antibiotics and esophageal disease, several types of studies are needed, including how antibiotics alter the esophageal microbiota, as well as functional studies of the role of various microbes in esophageal barrier function, inflammation, and carcinogenesis. Future studies regarding the effects of antibiotics and probiotics on esophageal integrity and cancer prevention should be prioritized because the knowledge gained may lead to new strategies to reduce the risk of esophageal malignancy.

### PPIs may alter gastric and esophageal microbiota

PPIs were introduced in the 1980s and dramatically change the gastric environment from a bactericidal low pH to a higher and more permissive pH. Indeed, in a small cohort of patients in whom gastric aspirates were cultured prior to starting a PPI, 10 of 12 patients had no growth from cultured gastric aspirate [32]. However, after starting PPIs, 80% of the patients without any initial (pre-PPI) growth developed positive gastric aspirate cultures [32]. Streptococci and *C*. *albicans* were the most frequently cultured organisms [32].

A separate study demonstrated that of control patients without PPI exposure, 21.3% had growth of non-*H*. *pylori* bacteria from gastric juice compared with 58.7% of those on PPIs [33]. Yet another study used esophageal biopsies to demonstrate marked changes in microbiota before and after PPI therapy with a decrease in *Comomonadaceae* and an increase in *Clostridia* and *Actinomycetales* after starting PPIs [34]. Finally, while limited to one patient as part of a longitudinal analysis of esophageal and gastric microbiota, this particular individual stopped PPI and experienced a marked shift in esophageal microbiota with decreased abundance of *Lactobacillus* and *Paralactobacillus*, suggesting that stopping PPIs may alter the esophageal microbiota [35]. While the effects of PPIs on the gastrointestinal microbiota are interesting (and PPI use should be controlled for in the analysis of clinical specimens), it is very important to note that the relationship of PPIs to specific alterations of the microbiota and how any PPI-mediated changes to the microbiota may or may not influence esophageal carcinogenesis remain unknown.

### Novel nonendoscopic approaches allow alternative methods of esophageal sampling

Many of the existing esophageal microbiome studies rely on endoscopic biopsy or brushing samples obtained during an upper endoscopy under sedation. The invasive nature of endoscopic biopsies and the cost of endoscopy remain a challenge, particularly given the global nature of esophageal cancer. Novel nonendoscopic approaches to esophageal sampling, including capsule and Cytosponge technology, have also been used to successfully assess the esophageal microbiome [36, 37]. In a comparative study between nasal swabs, oral esophageal string test (EST) samples, and mucosal biopsies, EST produced nearly identical bacterial profiles as mucosal biopsies [36]. However, some important differences were noted. In a comparison between mucosal biopsies and EST samples, *Pasteurella* was found in significantly higher relative abundance in the EST samples, while *Actinomyces* was found in greater relative abundance in mucosal biopsies. When comparing oral and EST samples, EST samples had higher amounts of *Prevotella* and lower amounts of *Neisseria* [36]. In another study comparing mucosal samples to biopsies, brush samples (typically taken during endoscopy) provided a superior yield of bacterial DNA and significantly improved the ratio of bacterial to host DNA, allowing detection of a greater number of bacterial taxa [35]. Alternatively, the Cytosponge is a nonendoscopic device that can be swallowed in capsule form, then expands within the stomach to be subsequently pulled back through the mouth. Although the Cytosponge has the advantage of being a nonendoscopic sampling method, yielding 10-fold higher quantities of microbial DNA than endoscopic brushes or biopsies, clustering analysis revealed that samples obtained using the Cytosponge cluster far away from biopsy or brush samples and likely represent dilutions from both gastric and oral bacteria [37]. As other nonendoscopic esophageal sampling methods such as the balloon are developed [38], larger cohorts will be needed to establish the role of noninvasive esophageal sampling methods for studies of the esophageal microbiota in conjunction with a potential role in early detection of BE, EAC, and ESCC [37].

## Discussion

Over the past few years, studies have strongly suggested that variation in the esophageal microbiota is associated with esophageal disease. Culture-based studies have revealed bacteria and yeast as important inhabitants of the esophageal microbiome. 16S rDNA studies have provided characterization of the bacteria in the esophagus and have identified an increase in gram-negative bacteria associated with GERD and esophagitis, BE, and EAC. However, the available studies are largely descriptive in nature with limited statistical analysis. Furthermore, given the cross-sectional nature of these studies, it is important to note that the reported association of gram-negative bacteria in the esophagus with esophagitis and BE is merely an association, and causality cannot be assumed; it is possible that esophagitis, BE, or esophageal cancer may create alterations in the esophageal mucosa that support changes in the esophageal microbiota. In addition, there are important limitations to many of the published studies on the esophageal microbiota. Many studies rely on “normal” samples obtained during endoscopy as controls; however, the patients undergoing endoscopy are rarely asymptomatic. In addition, the definitions of “esophagitis” and GERD vary between studies, making comparisons difficult. Finally, the overall quality of the existing literature must be considered. Recently, standards for analyses of the microbiota have been proposed [39, 40]. These standards suggest inclusion of positive controls (including known microbial species) and negative controls (because of high rates of contamination). Pei and colleagues clearly demonstrated that water-based negative controls yielded several species, and those were subtracted from the overall analysis [9]. Supporting Information S4 Table notes when controls were included in the methods, but overall, this group of early studies of the esophageal microbiota lacked the experimental rigor that one would expect today.

As methods for analyzing the microbiota evolve, we see a clear need for well-designed, broad, unbiased sequencing studies that include bacteria along with fungi and viruses in the esophagus. In addition, high-quality studies should account for clinical exposures that may change the esophageal microbiota. Medications, including antibiotics and PPIs, have been shown to alter the microbial ecosystem and thus may play important roles in provoking or preventing disease, but this remains an open area in need of further investigation. The potential role of PPIs in the pathologic alteration of the esophageal microbiome represents a particularly interesting area of research because while PPIs reduce acid levels (and acid-related esophageal damage), the higher pH leads to a less bactericidal milieu. The related shifts in the microbiota could actually be detrimental; however, conclusive studies in this area are still needed. The esophageal microbiome remains an exciting area for future research, particularly as relates to the functional role of particular organisms in metaplasia and carcinogenesis as well as how esophageal microbiota might be manipulated in order to favor esophageal health and prevent of metaplasia and cancer.

## Supporting information

S1 Table Pubmed MEDLINE database search strategy. (DOCX)

S2 Table Embase database search strategy. (DOCX)

S3 Table Web of Science database search strategy. (DOCX)

S4 Table Primary studies reporting the characterization of esophageal microbiota in normal and diseased esophagus. (DOCX)
