# Supplementary material for: Barrett’s esophagus and esophageal cancer: Links to microbes and the microbiome
Source: PLoS Pathog. 2018 Dec 20;14(12):e1007384. doi: 10.1371/journal.ppat.1007384 (PMC6301555; doi:10.1371/journal.ppat.1007384)
Supplement: S1 Table — (DOCX) [file ppat.1007384.s001.docx]

**Supporting Information**

**Database Search Strategy (search date: 2/9/2018)**

**S1 Table: Pubmed MEDLINE database search strategy**

| #1 | (“microbiota”[mesh] OR microbiome[tw] OR microbiota[tw] OR microflora[tw] OR “gastrointestinal flora”[tw] OR “gut flora”[tw] OR “intestinal flora”[tw] OR “enteric bacteria”[tw]) | 57563 |
| --- | --- | --- |
| #2 | (“Barrett esophagus”[mesh] OR “barrett’s esophagus”[tw] OR “barrett’s oesophagus”[tw] OR “barrett esophagus”[tw] OR “barrett oesophagus”[tw] OR esophagitis[tw] OR oesophagitis[tw]) | 25369 |
| #3 | (“esophageal neoplasms”[mesh] OR ((esophag*[tw] OR oesopha*[tw]) AND (neoplasm*[tw] OR cancer*[tw] OR “squamous cell”[tw] OR carcinoma*[tw] OR adenocarcinoma*[tw] OR tumor*[tw] OR tumour*[tw] OR dysplasia*[tw] OR “intestinal metaplasia”[tw]))) | 73771 |
| #4 | #2 OR #3 | 88764 |
| #5 | #1 AND 34 | 138 |

**Using all three databases (Medline, Embase and Web of Science):**

TOTAL CITATIONS BEFORE DUPLICATES REMOVED: 658

NUMBER OF DUPLICATE CITATIONS REMOVED IN ENDNOTE: 239

**TOTAL REFERENCES FOR MANUAL REVIEW: 419**
