# Supplementary material for: Barrett’s esophagus and esophageal cancer: Links to microbes and the microbiome
Source: PLoS Pathog. 2018 Dec 20;14(12):e1007384. doi: 10.1371/journal.ppat.1007384 (PMC6301555; doi:10.1371/journal.ppat.1007384)
Supplement: S2 Table — (DOCX) [file ppat.1007384.s002.docx]

**Supporting Information**

**Database Search Strategy (search date: 2/9/2018)**

**S2 Table: Embase database search strategy**

| #1 | ‘microflora’/exp OR microbiome:ab,ti OR microbiota:ab,ti OR microflora:ab,ti OR ‘gastrointestinal flora’:ab,ti OR ‘gut flora’:ab,ti OR ‘intestinal flora’:ab,ti OR ‘enteric bacteria’:ab,ti | 108,688 |
| --- | --- | --- |
| #2 | 'Barrett esophagus'/exp OR ‘barrett’s esophagus’:ab,ti OR ‘barrett’s oesophagus’:ab,ti OR ‘barrett esophagus’ab,ti OR ‘barrett oesophagus’:ab,ti OR esophagitis:ab,ti OR oesophagitis:ab,ti | 36654 |
| #3 | (‘esophagus tumor’/exp OR ((esophag*:ab,ti OR oesopha*:ab,ti) AND (neoplasm*:ab,ti OR cancer*:ab,ti OR ‘squamous cell’:ab,ti OR carcinoma*:ab,ti OR adenocarcinoma*:ab,ti OR tumor*:ab,ti OR tumour*:ab,ti OR dysplasia*:ab,ti OR ‘intestinal metaplasia’:ab,ti))) | 108709 |
| #4 | #2 OR #3 | 129956 |
| #5 | #1 AND #4 | 340 |

**Using all three databases (Medline, Embase and Web of Science):**

TOTAL CITATIONS BEFORE DUPLICATES REMOVED: 658

NUMBER OF DUPLICATE CITATIONS REMOVED IN ENDNOTE: 239

**TOTAL REFERENCES FOR MANUAL REVIEW: 419**
