# Supplementary material for: Barrett’s esophagus and esophageal cancer: Links to microbes and the microbiome
Source: PLoS Pathog. 2018 Dec 20;14(12):e1007384. doi: 10.1371/journal.ppat.1007384 (PMC6301555; doi:10.1371/journal.ppat.1007384)
Supplement: S3 Table — (DOCX) [file ppat.1007384.s003.docx]

**Supporting Information**

**Database Search Strategy (search date: 2/9/2018)**

**S3 Table: Web of Science database search strategy**

| #1 | Microbiome OR microbiota OR microflora OR “gastrointestinal flora” OR “gut flora” OR “intestinal flora” OR “enteric bacteria” | 73730 |
| --- | --- | --- |
| #2 | “Barrett esophagus” OR “barrett’s esophagus” OR “barrett’s oesophagus” OR “barrett oesophagus” OR esophagitis OR oesophagitis | 25895 |
| #3 | (esophag* OR oesopha*) AND (neoplasm* OR cancer* OR “squamous cell” OR carcinoma* OR adenocarcinoma* OR tumor* OR tumour* OR dysplasia* OR “intestinal metaplasia”) | 67685 |
| #4 | #2 OR #3 | 83967 |
| #5 | #1 AND #4 | 180 |

**Using all three databases (Medline, Embase and Web of Science):**

TOTAL CITATIONS BEFORE DUPLICATES REMOVED: 658

NUMBER OF DUPLICATE CITATIONS REMOVED IN ENDNOTE: 239

**TOTAL REFERENCES FOR MANUAL REVIEW: 419**
