# Supplementary material for: Barrett’s esophagus and esophageal cancer: Links to microbes and the microbiome
Source: PLoS Pathog. 2018 Dec 20;14(12):e1007384. doi: 10.1371/journal.ppat.1007384 (PMC6301555; doi:10.1371/journal.ppat.1007384)
Supplement: S4 Table — (DOCX) [file ppat.1007384.s004.docx]

**Supporting Information**

**S4 Table: Primary studies reporting the characterization of esophageal microbiota in normal and diseased esophagus.**

| **Year**  **Lead Author** | **Country** | **No. of Patients** | **Normal and/or Disease Type** | **Sampling Method** | **Method** | **Key Findings / Organisms Identified** | **Reference No** |
| --- | --- | --- | --- | --- | --- | --- | --- |
| 1982  Finlay | Scotland | 12 | 7 EAC 5 ESCC  (samples not separated by cancer type in analysis) | Surgical specimens | Culture | *Streptococcus (49% of total aerobic growth) and cultured in 11/12 patients*  *Bacteroides was also a common isolate in 11/12 patients* | ([9](#_ENREF_9)) |
| 1983  Mannell | South Africa | 101 | 51 surgical patients without esophageal disease  50 esophageal cancer (type unknown) | Intraoperative aspiration | Culture | *Streptococcus viridans* was common in both groups (74-88%)  Other frequent isolates:  *Haemophilus influenza* (24-37%)  *Streptococcus faecaliss* (30% of cancer patients)  *Neisseria catarrhalis* (18-24%)  Anaerobes:  *Peptococcus (67-88%)*  *Peptostreptococcus(42-63%)*  *Bacteroides melaninogenicus (24-43%)*  *Candida albicans* (present in 10%-12% of patients) | ([10](#_ENREF_10)) |
| 1998 Gagliardi | Brazil | 30 | Normal (endoscopy); non-specific symptoms) | Endoscopic aspiration | Culture | *Streptococcus viridans (30%)*  *Group D Streptococcus* (20%) | ([6](#_ENREF_6)) |
| 2004  Pei | USA | 4 | Normal histology with some symptoms prompting procedure | Endoscopic biopsy  Note that important controls were used, including a water only control in place of sample. Several organisms were identified and removed from reported esophageal results.* | rDNA sequencing | Normal Phyla (6):  *Firmicutes* 70% *Bacteroides* 20 % *Actinobacteria* 4%, *Proteobacteria* 2%, *Fusobacteria* 2%,  *TM7* 1%,  Note that most prevalent genera were  *Streptococcus* (39%)  *Prevotella* (17%)  *Veillonella* (14%) | ([11](#_ENREF_11)) |
| 2005 Pei | USA | 24 | 9 normal  12 GERD – defined as symptoms and histologic findings of GERD: “mucosal erosions/superficial ulcerations, epithelial hyperplasia, and inflammatory infiltrate of polymorphonuclear cells or eosinophils in the mucosal layer”  3 BE | Endoscopic biopsy | Histology and  rDNA sequencing | They concluded that diverse and complex bacterial populations were present in the distal esophagus; in 48 clones from 24 patients they identified:  *Prevotella veroralis* 44% normal,  8% GERD  *Streptococcal species* 11% normal, 17% GERD  *Pseudomonas species* 17% GERD,  33% BE  Oral bacterium SH66  67% normal,  25% GERD,  67% BE | ([12](#_ENREF_12)) |
| 2007 Macfarlane | Scotland | 14 | 7 endoscopically and histologically normal  7 BE | Endoscopic biopsy and aspirate | Histology and Culture with rDNA sequencing of cultured isolates | Lactobacilli, streptococci and yeast were detected in aspirates from normal esophagus  and BE.  When pH was <2, no bacteria were visible.  BE patients had greater diversity and gram negative cocci were identified in mucosa.  57% of BE patients had *Campylobacter*, but none was present in normal esophagus. | ([13](#_ENREF_13)) |
| 2009 Yang | USA | 34 | 12 histologically normal samples (two of these had BE and/or tumor adjacent to the normal sample)  12 with esophagitis on histology**  10 BE | Endoscopic biopsy | rDNA sequencing | The “normal”, *Streptococcus* predominant type matched 91.7% of the normal samples.  The “diseased” esophagus included more gram-negative anaerobes and “microaerophiles” and this diseased profile matched 58.3% esophagitis and 60% BE samples. | ([14](#_ENREF_14)) |
| 2012 Fillon | USA | 15 | 15 children ages 7-20 undergoing endoscopy for symptoms with normal histology | Esophageal String Test (EST) followed by upper endoscopy | rDNA sequencing  included negative controls in PCR experiments | Most common genera (by biopsy, EST):  *Streptococcus* 39%, 41%,  *Provotella* 16%, 23%,  *Veillonella* 7%, 9%  were the most common genera identified. | ([15](#_ENREF_15)) |
| 2013 Liu | Japan | 18 | 6 endoscopically and histologically normal samples  6 reflux esophagitis  6 BE | Endoscopic biopsy | rDNA sequencing  For quantification, positive controls were included in RNase-free water for generation of standard curves | Normal subjects: *Proteobacteria* 49%  *Firmacutes* 40%  *Bacteroidetes* 8%  *Actinobacteria* 3%  GERD:  *Proteobacteria* 43%  *Firmicutes* 33%  *Bacteroidetes* (10%)  *Fusobacteria* (10%)  *Acitinobacteria* 2%  *TM7* 2%  BE:  *Firmacutes* 50% (with loss of *Streptococcus*, but increase in *Veillonella*)  *Proteobacteria* 20%  *Bacteroidetes* 14% (*Prevotella*)  *Fusobacteria* 9%  *Actinobacteria* 2% | ([16](#_ENREF_16)) |

| 2013 Blackett | UK | 131 | 39 normal (iron deficiency as presenting indication for endoscopy) with normal endoscopy and histology and no reflux symptoms  37 GERD with endoscopic evidence of esophagitis  45 BE  30 EAC | Endoscopic biopsy | Culture (including culture conditions for campylobacter)  and rRNA sequencing | *Campylobacter* (mostly *C. Concisus*) increased in association with GERD and BE  *C. Concisus* present in:  13% normal,  51% GERD,  42% BE, and  9% EAC  Increase in IL-18 in GERD and BE colonized by campylobacter | ([17](#_ENREF_17)) |
| --- | --- | --- | --- | --- | --- | --- | --- |
| 2013 Grusell | Sweden | 40 | 40 normal esophageal samples from patients without gastrointestinal symptoms undergoing head and neck surgical procedures. | Endoscopic biopsy and brushing | Culture | Most prevalent cultured microbe was *Streptococci viridans*  Similar occurrence rate in brush and biopsy samples (98% and 95% respectively) | ([7](#_ENREF_7)) |

| 2014 Amir | Israel | 34 | All patients had heartburn symptoms:  15 normal samples with GERD symptoms  13 esophagitis  6 BE | Endoscopic biopsies and gastric fluid | rDNA sequencing | Gastric fluid showed increased *Enterobacteriacieae* in esophagitis and BE (30%) vs GERD without esophagitis (6%)  Microbiota of esophageal biopsy samples and gastric fluid changed after PPI treatment | ([18](#_ENREF_18)) |
| --- | --- | --- | --- | --- | --- | --- | --- |
| 2015  Gall | USA | 12 | 12 BE  (83% on acid suppression)  3 patients with samples from two time points (BE surveillance) | Endoscopic biopsies and brushings | rDNA sequencing | Brushing the mucosa yielded all mucosal organisms found in biopsies as well as additional organisms  Most prevalent phyla:  *Firmacutes*  *Bacteroidetes*  Most prevalent genera:  *Streptococcus* and *Prevotella* | ([19](#_ENREF_19)) |
| 2016  Zaidi | USA | 28 | 3 tumor-adjacent normal epithelium  13 BE  3 Dysplasia  5 EAC  8 GERD samples of normal-appearing esophagus (no esophagitis) | Snap frozen human samples | PCR/FISH  PCR plate included “internal calibrator of synthetic DNA” | Most prevalent organism detected was Candida sp. 30% all human samples  *Candida albicans* and *Candida glabrata* was detected in >50% of EAC samples  *Streptococcus was present in 67% human samples.*  *Escherichia coli* was detected in BE and EAC samples | ([20](#_ENREF_20)) |
| 2016  Yamamura | Japan | 326 | 300 SCC  12 EAC  13 other | Surgical specimen | qPCR | *Fusobacterium nucleatum* was present in 23% esophageal cancer with more DNA in cancer vs non-tumor tissues  (p-value 0.02) | ([21](#_ENREF_21)) |
| 2017 Peters | USA | 106 | 81 EAC  25 ESCC | Oral | rDNA sequencing  DNA extraction and sequencing was performed in one lab and was blinded with technical replicates | Peridontal pathogen *Tannerella forsythia* associated with increased EAC risk 1.21 (95% CI 1.01-1.46), p-value 0.04 | ([22](#_ENREF_22)) |

* Pei et al (2004) ([11](#_ENREF_11)) included an important water-only control. The following organisms were isolated: *Pseudomonas tolaasii*, *Pseudomonas influorensces*, *Pseudomonas syringae*, *Pseudomonas putida*, uncultured *Duganella* clone CTHB-18 (AF067655), *Stenotrophomonas maltophilia*, *Janthinobacterium lividum*, *Lactobacillus paracasei*, *Propionibacterium* *acnes*, *Pseudomonas antarctica/meridiana*, and *Brevundimonas bulata* were all isolated by Pei et al (2004) from water controls and subtracted from the esophageal biopsy results.

** Yang et al 2009, defined esophagitis as: ≥ 10 lymphocytes per high powered field, any number of eosinophils or polymorphonuclear cells
